# Digital Health Portals for Individuals Living With or Beyond Cancer: Patient-Driven Scoping Review

**DOI:** 10.2196/72862

**Published:** 2025-07-18

**Authors:** Steven Ouellet, Florian Naye, Wilfried Supper, Chloé Cachinho, Marie-Pierre Gagnon, Annie LeBlanc, Marie-Claude Laferrière, Simon Décary, Maxime Sasseville

**Affiliations:** 1 Faculté des sciences infirmières, Université Laval Québec, QC Canada; 2 École de réadaptation, Faculté de médecine et des sciences de la santé, Université de Sherbrooke Sherbrooke, QC Canada; 3 Centre de recherche en santé durable Vitam Québec, QC Canada; 4 Département de médecine de famille et de médecine d'urgence, Faculté de médecine, Université Laval Québec, QC Canada; 5 Bibliothèque, Université Laval Québec, QC Canada

**Keywords:** cancer, oncology, patient portal, electronic health records, online access, patient records, social determinants of health, scoping review, Preferred Reporting Items for Systematic Reviews and Meta-Analyses

## Abstract

**Background:**

Digital health portals are online platforms allowing individuals to access their personal information and communicate with health care providers. While digital health portals have been associated with improved health outcomes and more streamlined health care processes, their impact on individuals living with or beyond cancer remains underexplored.

**Objective:**

This scoping review aimed to (1) identify the portal functionalities reported in studies involving individuals living with or beyond cancer, as well as the outcomes assessed, and (2) explore the diversity of participant characteristics and potential factors associated with portal use.

**Methods:**

We conducted a scoping review in accordance with the JBI methodology (formerly the Joanna Briggs Institute) and the PRISMA-ScR (Preferred Reporting Items for Systematic Reviews and Meta-Analyses extension for Scoping Reviews) guidelines. We included primary research studies published between 2014 and 2024 that involved participants living with or beyond cancer, had access to personal health information, and assessed at least one outcome related to health or the health care system. We searched the Embase, Web of Science, MEDLINE (Ovid), and CINAHL Plus with Full Text databases. Five reviewers independently screened all titles, abstracts, and full texts in duplicate using Covidence. We extracted data on study design, participant characteristics, portal functionalities, outcomes assessed, and PROGRESS-Plus (place of residence; race, ethnicity, culture, or language; occupation; gender or sex; religion; education; socioeconomic status; and social capital–Plus) equity factors.

**Results:**

We included 44 studies; most were conducted in the United States (n=30, 68%) and used quantitative (n=23, 52%), mixed methods (n=11, 25%), or qualitative (n=10, 23%) designs. The most common portal features were access to test results (28/44, 64%) and secure messaging (30/44, 68%). Frequently reported services included appointment-related functions (19/44, 43%), educational resources (13/44, 30%), and prescription management features (11/44, 25%). Behavioral and technology-related outcomes were the most frequently assessed (37/44, 84%), followed by system-level (19/44, 43%), psychosocial (16/44, 36%), and clinical outcomes (5/44, 11%). Overall, 43% (19/44) of the studies addressed PROGRESS-Plus factors. Age was the most frequently reported (13/19, 68%), followed by socioeconomic status (10/19, 53%), race or ethnicity (7/19, 37%), and gender or sex (7/19, 37%). Social capital (2/19, 11%), occupation (1/19, 5%), and disability (1/19, 5%) were rarely considered, and religion was not reported in any study.

**Conclusions:**

While digital health portals enhance patient engagement, their clinical impact and equity implications remain insufficiently evaluated. We found disparities in functionalities, outcomes, and PROGRESS-Plus representation. To promote equitable benefits, future studies should adopt inclusive designs and evaluation strategies that address diverse outcomes and integrate social determinants of health.

## Introduction

### Background

Patient portals are digital platforms designed to improve health outcomes and the quality of care by facilitating health data access and communication between individuals and their health care providers [1-5]. These portals offer remote access to provider-owned personal medical records from any location with internet connectivity [1,3-7]. By enabling timely communication with care teams and supporting informed decision-making, portals have the potential to enhance disease-related knowledge and patient engagement, while also contributing to the optimization of health care processes [1-5,7-11]. Their growing use reflects a shift toward empowering individuals and supporting more active and collaborative approaches to health management [12-16].

For conceptual precision, the terminology used in this study aligns with definitions commonly found in the peer-reviewed literature. Although similar in function, personal health records (PHRs) and patient portals differ in several ways [9,10,17]. PHRs are personally owned and controlled tools that allow individuals to enter, manage, and integrate health data from multiple sources. In contrast, patient portals are institutionally managed and contain information from one or more health care providers [9,10,17]. While PHRs generally provide greater user autonomy and integration of personal health information, patient portals are typically tethered to health care providers systems to facilitate interoperability [9,10,17].

Distinguishing electronic health records (EHRs) from electronic medical records (EMRs) is also relevant. EHRs are comprehensive, provider-maintained digital records intended for use across health care systems to support coordinated care and clinical decision-making [17]. Patient portals, in contrast, offer individuals limited access to selected health information contained within these systems, such as laboratory or tests results [7,17]. Although similar, EHRs differ from EMRs in scope [12]. EMRs function as digital equivalents of paper charts, typically limited to a single practice, whereas EHRs integrate information across multiple providers and support greater interoperability [7,11,17].

Patient portals, tethered to EHRs or EMRs, are secure online platforms enabling individuals to access their personal administrative and clinical information at any time and from any location [1,2]. This access to personal health information constitutes the core functionality of digital health portals, regardless of whether they are referred to as patient portals or PHRs [1,2,8]. More recent generations of portals can also include interoperable features that facilitate communication and care coordination with health care providers, such as secure messaging, appointment scheduling, and medications renewal capabilities [1,2].

Cancer care presents both challenges and opportunities for the implementation and meaningful use of these capabilities [3,4,6,8]. The complexity of oncology care, involving multidisciplinary teams, intensive treatments, and frequent clinical interactions, highlights the need for effective information management and communication systems. Patient portal can improve communication in complex context by promoting informational continuity, enhancing care coordination, and supporting engagement among individuals living with or beyond cancer [1-8]. In addition to (1) accessing their personal health information, these benefits are supported by enabling individuals to (2) communicate with providers through secure messaging and (3) access health services [1,2,4-7].

Improvements in health outcomes, including enhanced disease-related knowledge and self-efficacy, were associated with portal use for the chronic disease management contexts [1,5,7]. For instance, in diabetes management, portal use has been associated with improved clinical outcomes such as better glycemic control [1]. Evidence regarding clinical benefits in oncology, however, remains inconclusive. Studies focusing on breast cancer populations have demonstrated no consistent relationship between portal use and improvements in symptom management [8]. In addition, portals may contribute to improved health system efficiency by decreasing wait times and reducing missed appointments [2]. Research on their impact on health care use within diabetes management remains limited [1].

Patient portals and PHRs are associated with a range of potential benefits, spanning behavioral changes and system-level efficiencies [1,2,5,7,12-14,16,18,19]. A comprehensive assessment of the impact of digital health technologies requires consideration of multiple outcome domains [20,21]. These include behavioral and technology-related outcomes (eg, self-management, health behaviors, usability, and perceived usefulness); psychosocial outcomes (eg, emotional well-being and quality of life); clinical outcomes (eg, symptom burden, fatigue, and nutritional status); and system-level outcomes (eg, care coordination, cost-effectiveness, and hospital readmissions) [20]. However, substantially gaps remain in evaluating patient portals across multiple dimensions, along with limited understanding of the full range of outcomes associated with their use [21].

The use and impact of portals across diverse population groups remain insufficiently explored [22]. The PROGRESS-Plus (place of residence; race, ethnicity, culture, language, or occupation; gender or sex; religion; education; socioeconomic status; and social capital–Plus) framework offers a comprehensive lens for examining these disparities by highlighting social determinants of health [23]. For example, individuals in rural areas may face limited internet access, while patients from racial or ethnic minority groups may have lower rates of portal adoption. Socioeconomic constraints, lower educational attainment, and reduced social support have also been associated with possible decreased portal use [3-6,22,24,25]. Integrating the PROGRESS-Plus framework into evaluations of portal use in oncology may support the identification of inequities and inform the development of more inclusive digital health strategies.

### Objectives

This scoping review aimed to identify the digital health portal functionalities reported in studies involving individuals living with or beyond cancer, as well as the categories of health outcomes assessed, including those related to the health care system. A secondary objective was to explore the diversity of participant characteristics and potential factors associated with portal use.

## Methods

### Overview

Aligned with the Canadian Institutes of Health Research Strategy for Patient-Oriented Research and Patient Engagement Framework [26], this study actively engaged “patient partners” (SO and CC), who are also coauthors, throughout all phases of the project. The Canadian Institutes of Health Research defines “patient partners” as individuals with lived experience of a health condition who engage meaningfully in the research process as members of the study team. In this review, SO and CC contributed to shaping the research objectives, codeveloping the work plan and study protocol with the full author team, and participating in the interpretation of findings.

This scoping review was conducted in accordance with the JBI (formerly the Joanna Briggs Institute) guidelines [27], and the protocol was registered in the Open Science Framework Registries [28]. The results are reported following the PRISMA-ScR (Preferred Reporting Items for Systematic Reviews and Meta-Analyses extension for Scoping Reviews) checklist [29]. The PCC (population [or participant], concept, and context) framework [30,31] was used to define the elements applied in this review (Table 1).

**Table 1:** Inclusion and exclusion criteria, study designs, and study types.

PCC^a^ elements [31], study designs, and study types	Inclusion criteria	Exclusion criteria
Population	Individuals living with or beyond cancer, including children, teenagers, and adults, as well as their informal caregivers or family members	Mixed groups of cancer and noncancer participants when subgroup-specific results for participants with cancer were not reported Breast, prostate, or lung cancer screening studies involving populations without a formal cancer diagnosis Studies focused solely on clinicians’ perceptions or the impact on their workload (clinician-only studies)
Concept	Access to personal health information on a digital portal At least one outcome related to health, or the health care system assessed	Use of a digital portal for a specific purpose, such as surveying patients on a topic unrelated to the portal itself Studies only addressing usability tests or portal development outcomes
Context	At home or in another outpatient setting	Hospitalized
Study design and study type	Randomized controlled trials, quasi-randomized controlled trials, prospective cohort studies, pretest-posttest studies, observational studies, mixed methods studies, qualitative studies, and quantitative descriptive (surveys presenting participant characteristics)	Reviews, opinions, editorials, commentaries, book chapters, and conference papers

^a^PCC: population (or participant), concept, and context.

### Search Strategy

The initial development of the search strategy was informed by 2 previously published systematic reviews: one examining patient portals functionalities and health outcomes in individuals with diabetes [1] and the other focusing on eHealth technologies for supportive care in breast cancer [8]. A preliminary search was first developed by the first author (SO) and the corresponding author (MS), drawing on the approaches used in these reviews. This strategy was subsequently refined in collaboration with an experienced librarian (MCL), who provided guidance on the final search terms and structure.

The search was conducted across 4 databases: Embase; Web of Science (SCI-EXPANDED, SSCI, AHCI, and ESCI); MEDLINE (Ovid); and CINAHL Plus with Full Text (EBSCOhost) to identify sources published between January 1, 2014, and February 27, 2024. Overall, 10 relevant sources, identified through hand-searching by the first author (SO), were used to assess the sensitivity of the database-specific search strategies provided in Multimedia Appendix 1. References were imported into the web-based collaborative tool Covidence [32] by the librarian (MCL), where duplicates were removed using both manual verification and the platform’s automated deduplication function.

The search start date was restricted to 2014 to ensure the relevance of the findings to contemporary technological capabilities. The past decade has seen rapid advancements in digital health, particularly in the adoption of patient portals and the availability of enhanced features [14,18,19]. Reflecting the fast-paced evolution of eHealth research, one review limited its search to studies published from 2016 onward [8]. In the United States, more recent generations of portals began gaining traction around 2012, with broader adoption and increasing research interest by 2015 [18]. In addition to providing access to laboratory and tests results, these portals increasingly incorporated functionalities such as secure messaging, prescription renewals, and appointment scheduling, contributing to more patient-centered and interoperable systems [14,19].

### Data Collection

As shown in Table 1, the inclusion criteria were (1) participants living with or beyond cancer, (2) access to personal health information through a digital portal, and (3) at least one outcome related to health or the health care system. Studies conducted in hospital settings were excluded, as patients with cancer in these environments are typically managed by clinical teams overseeing all aspects of care and support. In addition, studies involving mixed populations of cancer and noncancer participants were excluded if subgroup-specific results for individuals living with or beyond cancer were not reported.

To ensure consistency in the application of the eligibility criteria, a calibration exercise was conducted before the screening phase. A sample of 20 records was independently reviewed by 5 team members (SO, WS, CC, FN, and MS), including one experienced reviewer (MS). During this process, it was observed that some titles and abstracts referred to access to EHRs or PHRs rather than explicitly using the term “patient portal.” Regardless of terminology, inclusion or exclusion decisions were based strictly on alignment with the predefined selection criteria.

Following calibration, the same 5 reviewers screened all titles and abstracts in duplicate using the established criteria. Discrepancies regarding inclusion at this stage were resolved through group consensus. Before full-text screening, a second calibration exercise was performed using a sample of 10 articles to further ensure consistency. Full-text review was also conducted in duplicate by the same team, with any disagreements regarding study inclusion resolved through consensus among all reviewers.

### Data Extraction

In accordance with JBI guidance [33], a structured data extraction grid was developed and pretested during a team meeting involving all reviewers. Four reviewers (WS, CC, SO, and FN) independently extracted data from the included studies, and the results were subsequently verified by the first author (SO) and an experienced reviewer (MS) to ensure accuracy and completeness. A Microsoft Excel spreadsheet was used to manage the data extraction process. Extracted information included general study characteristics (such as article reference, first author, year of publication, country, study method, data source, and participant characteristics) and portal-related details (including portal name, type of accessible health information, availability of secure messaging, and access to health services provided), and reported outcomes.

### Data Synthesis

All included studies involved portal use, defined as participants having access to their personal health information through a digital platform [1,2,4-7]. This include both patient portals and PHRs [9]. Data synthesis was structured using 3 conceptual frameworks. First, portal features were classified into three categories: (1) type of accessible health information, (2) availability of secure messaging, and (3) access to health services through the portal [1,2,7].

Second, study outcomes were grouped into four domains: (1) behavioral and technology-related experiences, (2) psychosocial outcomes, (3) clinical outcomes, and (4) health care system–related outcomes [20,28].

Third, the PROGRESS-Plus framework was applied to identify dimensions of social stratification that may influence portal use and related outcomes [23]. This framework includes the following factors: place of residence, race or ethnicity, occupation, gender or sex, religion, education, socioeconomic status, and social capital. The “Plus” component captures additional sources of potential disadvantage, such as age, disability, and other vulnerabilities relevant to health equity.

## Results

### Overview

**Figure 1:**
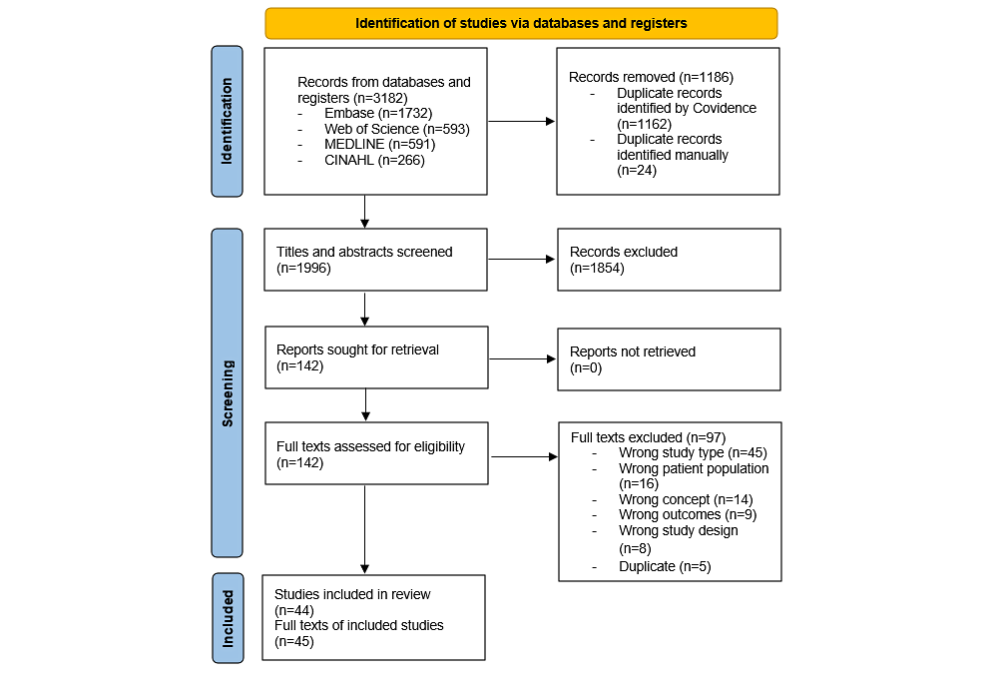
PRISMA 2020 flow diagram.

Out of 1996 titles and abstracts, along with 142 full-text articles that underwent dual screening, 44 studies reported across 45 articles (1 study was reported in 2 separate articles) met the eligibility criteria. The PRISMA (Preferred Reporting Items for Systematic Reviews and Meta-Analyses) 2020 flow diagram is shown in Figure 1 [34].

### Characteristics of Included Studies

The characteristics of the included studies are presented in Table 2.

**Table 2:** Characteristics of included studies.

Study; country	Study type, method, and data source	Participants (number and characteristics)
Alpert et al [35], 2019; United States	Qualitative Interviews	35 patients with cancer, 13 oncologists, and 12 informaticists
Baun et al [36], 2020; Denmark	Mixed methods Questionnaires and interviews	Questionnaires: 46 patients with cancer Interviews: 4 patients with cancer
Cahill et al [37], 2014; United States	Quantitative descriptive Data from different sources	186 patients with cancer
Colussi et al [38], 2024; Argentina	Qualitative descriptive Free text field in a survey	422 survey responses; possible duplicate responses from patients with cancer
Conroy et al [39], 2023; United States	Quantitative descriptive Data from the electronic medical records	4069 patients with cancer
Coquet et al [40], 2020; United States	Quantitative descriptive Data from the electronic health records	9900 patients with cancer (6446 patients after propensity score matching)
Daly et al [41], 2020; United States	Mixed methods Single-arm pilot study Questionnaire and interviews	100 patients with cancer.
DeRegge et al [42], 2020; Belgium	Mixed methods Survey, interviews, and logged data	23 patients with cancer
Ector et al [43], 2020; Netherlands	Qualitative Pilot study Interviews	8 patients with cancer
Elkefi et al [44], 2021; United States	Quantitative descriptive Survey	Total patients: 4328 With cancer: 683
Emamekhoo et al [45], 2023; United States	Quantitative. Questionnaire.	2076 patients with cancer
Fridriksdottir et al [46], 2023; Iceland	Mixed methods Questionnaire and interviews	69 patients with cancer
Geerts et al [47], 2023; Netherlands	Mixed methods Questionnaire and interviews	204 patients with cancer
Geerts et al [48], 2019; Netherlands	Mixed methods Questionnaire and interviews	18 patients with cancer
Gerber et al [49], 2014; United States	Quantitative Data from the patient portal	6495 patients with cancer
Greenberg-Worisek et al [50], 2020; United States	Quantitative descriptive Secondary analysis of survey data	3031 patients with or beyond cancer (“survivors”)
Griffin et al [51], 2024; United States	Quantitative descriptive Data from the patient portal	28,942 patients with cancer
Groen et al [52], 2017, Netherlands	Mixed methods Questionnaires, a focus group, and analysis of user log data	37 patients with cancer
Haggstrom and Carr [53], 2022; United States	Qualitative Interviews	6 patients with cancer and 4 caregivers
Kayastha et al [54], 2018; United States	Qualitative Interviews	20 patients with cancer
Kuijpers et al [55], 2016; Netherlands	Mixed methods Questionnaire and focus group	92 patients with cancer
Leader et al [56], 2021; United States	Quantitative Survey of patients and caregivers	346 patients with cancer and 13 caregivers
Liu et al [57], 2022; United States	Quantitative Survey	626 patients with cancer
Longacre et al [58], 2023; United States	Mixed methods Data from the portals, surveys, and interviews	20 patients with cancer and 19 caregivers
Luo et al [59], 2022; United States	Quantitative descriptive Survey	207 patients with cancer
Luoh et al [60], 2021; United States	Quantitative A retrospective analysis of portal use data	5950 patients with cancer
McCleary et al [61], 2018; United States	Mixed methods Survey and focus groups	Survey: 1019 patients with cancer; focus groups: 20 staff, 5 patients
Nahm et al [62], 2019; United States	Quantitative Questionnaire	30 patients with cancer
Ngo et al [63], 2020; United States	Qualitative Interviews	27 patients with cancer
O’Connor et al [64], 2022; United Kingdom	Mixed methods Service utilization data, online surveys, and interviews.	518 patients with cancer
Pho et al [65], 2019; United States	Quantitative Data from the “MyChart” portal log-in records	2524 patients with cancer
Rexhepi et al [66,67], 2018, 2021; Sweden	Qualitative Interviews	30 patients with cancer
Rexhepi et al [68], 2020; Sweden	Quantitative Survey	Total patients: 2587 With cancer: 347
Santos et al [69], 2021; Canada	Qualitative Interviews	10 patients with cancer and 1 family caregiver
Schultz and Alderfer [70], 2018; United States	Qualitative Interviews	19 caregivers of children with cancer
Schultz et al [71], 2021; United States	Quantitative descriptive Data from a hospital database	390 caregivers of children with cancer
Shaverdian et al [72], 2019; United States	Quantitative Survey	136 patients with cancer (baseline survey completed)
Strekalova [73], 2019; United States	Quantitative Survey	542 patients with cancer
Tarver et al [74], 2019; United States	Quantitative Survey	22 patients with cancer
Vachon et al [75], 2022; United States	Quantitative Survey	22 patients with cancer
Weis et al [76], 2020; Germany	Qualitative Interviews	22 patients with cancer and 9 caregivers
Wickersham et al [77], 2019; United States	Quantitative Survey	85 patients with cancer
Williamson et al [78], 2017; United States	Quantitative descriptive Data from the medical charts	624 patients beyond cancer (“survivors”)
Wolff et al [79], 2019; United States	Quantitative pilot randomized controlled trial Surveys	132 patient and family caregiver dyads

The 45 included articles (reporting on 44 studies) were published between 2014 and 2024, with an increase beginning in 2018 (4/45, 9%) compared to 2017 (2/45, 4%) [35-79]. The highest number of publications was observed in 2019 (9/45, 20%) and 2020 (9/45, 20%), followed by a decline in 2021 (6/45, 13%) and 2022 (6/45, 13%). This distribution is presented in Multimedia Appendix 2.

Of the 44 included studies, most were conducted in the United States (30/44, 68%), followed by the Netherlands (5/44, 11%) and Sweden (2/44, 5%). Seven other countries were each represented by a single study. Most studies used a quantitative design (23/44, 52%), followed by mixed methods (11/44, 25%) and qualitative approaches (10/44, 23%).

The number of participants with cancer ranged from 6 to 6495, or 9900 (reduced to 6446 after propensity score matching) in one study. Informal or family caregivers were included in 18% (8/44) of the studies, while individuals beyond active cancer treatment, described as cancer survivors, were included in 5% (2/44) of the studies.

### Cancer Types and Stages

The cancer types and stages of participants in all included studies are presented in Multimedia Appendix 3. Among the 44 included studies, breast cancer was the most frequently reported cancer type (16/44, 36%), followed by hematologic cancers, including leukemia, lymphoma, and multiple myeloma (12/44, 27%). Gastrointestinal cancers, such as colorectal and stomach cancers, were reported in 18% (8/44) of the studies. Lung cancer was reported in 18% (8/44) of the studies, and prostate cancer was reported in 16% (7/44) of the studies. Sarcomas were reported in 11% (5/44) of the studies, brain tumors in 5% (2/44) of the studies, and kidney cancer in 5% (2/44) of the studies. Metastatic disease was identified among participants in 25% (11/44) of the studies, often involving advanced stages, including stage IV.

### Portal Functionalities Reported and Outcomes Assessed

All 44 included studies involved participants who had access to their personal information or data through a digital portal. However, access to secure messaging features or other health services was not a required for inclusion. To address the primary objective of this review, which was to identify the functionalities of portals used by individuals with cancer and the outcomes assessed, their characteristics of are presented in Table 3.

**Table 3:** Portal functionalities and outcomes assessed.

Study; country	Portal name and type of accessible health information	Availability of secure messaging	Access to health services provided	Assessed outcomes
Alpert et al [35], 2019; United States	Web-based apps that provide 24×7 access to EMRs^a^ Laboratory tests results and imaging reports	Yes	Appointment scheduling, medication refills	Oncologist-patient communication Patient engagement in their care and potential anxiety
Baun et al [36], 2020; Denmark	“Patient-accessible electronic health record” Medical records, scan reports, laboratory results, and medication lists	Not mentioned or unrelated to the study objective	Not mentioned or unrelated to the study objective	Patients’ attitudes and experiences with online access to scan results
Cahill et al [37], 2014; United States	“MyMDAnderson,” the patient portal at MD Anderson Cancer Center Physician notes, surgical reports, laboratory results, pathology reports, and diagnostic imaging reports	Yes	Appointment scheduling, medication refills, and educational resources	How portal use correlates with disease-related uncertainty, symptom severity, and mood
Colussi et al [38], 2024; Argentina	“Mi Portal,” the patient portal at Instituto Alexander Fleming Clinical and administrative information (to be integrated)	Not mentioned or unrelated to the study objective	Appointment scheduling (the portal’s initial functionality)	Patient needs and expectations: access to clinical and administrative information, communication, and preparation for treatments.
Conroy et al [39], 2023; United States	Epic MyChart patient portal Medical history, test results, and clinical notes	Yes	Appointment scheduling, medication refills, and health questionnaires	Racial and ethnic differences in messaging use among patients with breast cancer
Coquet et al [40], 2020; United States	“MyHealth” patient portal at Stanford Cancer Institute Medical history, test results, and clinical notes	Yes	Appointment scheduling and medication refills	2-year survival in patients undergoing chemotherapy by patient portal email use
Daly et al [41], 2020; United States	The “Memorial Sloan-Kettering” patient portal Access to a digital, remote symptom management system	Yes; in addition to secure messaging, remote consultations are available through the portal	Electronic symptom tracking with real-time alerts and trend monitoring	Patient engagement, frequency of symptom alerts, and perceived value Likelihood of using acute care
DeRegge et al [42], 2020; Belgium	The “Digital Oncology Platform” integrated into the “Flanders Collaborative Care Platform” Laboratory results, discharge letters, and research reports	Yes	Appointment scheduling; personalized symptom tracking, education, and care planning via an online platform	Patient adoption, usability, and provider engagement
Ector et al [43], 2020; Netherlands	“CMyLife,” a web-based platform designed to support patients with chronic myeloid leukemia Access logs of symptoms and laboratory results, including molecular marker tracking	Yes; virtual consultations enable direct patient-provider communication	Integrated platform for symptom tracking, medication management, personalized feedback, and patient education	Impact of “CMyLife” on self-management, guideline adherence, and hospital visits
Elkefi et al [44], 2021; United States	Overview of online medical records and patient portals Study on patient portal use barriers and demographic adoption trends among patients with cancer	Not mentioned or unrelated to the study objective	Not mentioned or unrelated to the study objective	Factors influencing use of portals: demographic disparities, privacy concerns, and preference for communication with health care providers.
Emamekhoo et al [45], 2023; United States	“Epic MyChart” patient portal Access to test results and personal medical history	Yes	Medication review and access to appointment history	Log-in frequency, appointment proximity, functionality use, and demographic differences
Fridriksdottir et al [46], 2023; Iceland	Portal integrated within the Icelandic EMR system Symptom monitoring system for patient health tracking	Yes	Symptom and distress monitoring with alerts, educational materials, and targeted follow-up	Portal feasibility: adoption, usability, symptom improvement, and health engagement
Geerts et al [47], 2023; Netherlands	“MM E-coach,” an eHealth application designed to support patients during treatment Medication management (to be implemented)	Not yet (to be implemented)	Not yet (to be implemented)	Patient preferences, physician concerns, and main portal engagement factors such as communication tools, timing of access, and security considerations
Geerts et al [48], 2019; Netherlands	“MM E-coach,” an eHealth application designed to support patients during treatment An overview of prescribed medications, including dosage, frequency, and reminders, with the option for patients to register their intake	Yes	PRO^b^ assessments track symptoms and well-being, alerts notify of severe symptoms, a personalized care plan sets and tracks treatment goals, educational resources, treatment options, and supportive care	Usability (system usability scale), patient engagement, and messaging service use
Gerber et al [49], 2014; United States	“Epic MyChart” patient portal Patient access to test results and personal health records	Yes	Appointment scheduling, medication renewals, health library access, and billing information	Predictors and patterns of “MyChart” use among patients with cancer, including adoption, use frequency, common actions, and demographic trends
Greenberg-Worisek et al [50], 2020; United States	Overall “Electronic Personal Health Information Tool” (ePHI) tool use Reviewing test results	Yes	Tracking medical appointments and managing health care–related paperwork	Differences in ePHI use and email communication with providers between rural and urban patients with cancer
Griffin et al [51], 2024; United States	Overview of EHR^c^-linked patient portals across health care systems Access to laboratory and imaging results	Yes	Tools for appointment management and medication review	Disparities in portal access, use persistence, and barriers
Groen et al [52], 2017; Netherlands	“MyAVL,” an interactive patient portal developed for patients with lung cancer at the Netherlands Cancer Institute Patient access to blood tests, physiological results, pathology reports, and physician notes	Not mentioned or unrelated to the study objective	Patient education, appointment overview, PROs feedback, and personalized activity support	Patient satisfaction, perceived value, and sense of control; impact on activation, quality of life, or physical activity
Haggstrom and Carr [53], 2022; United States	The “OpenMRS” medical record system platform, an open-source software for managing medical records Cancer diagnosis, treatment overview, and tracking of recommended and completed surveillance tests	Yes	Self-management guidance, support group links, controlled access for caregivers and providers, and personal reflections in a dedicated journal	Stakeholder perspectives (patients, caregivers, and providers) on the usability, access, and implementation of the PHRc, exploring its impact on self-management, communication, and workflow integration
Kayastha et al [54], 2018; United States	“Epic MyChart” patient portal Patient access to oncology notes, medical history, test results, and treatment plans	Not mentioned or unrelated to the study objective	Not mentioned or unrelated to the study objective	How reading clinician notes impacts patient comprehension, trust, anxiety, and engagement in cancer care.
Kuijpers et al [55], 2016; Netherlands	“MijnAVL,” an interactive patient portal developed for breast cancer survivors Patient access to laboratory, pathology, and radiology results, multidisciplinary meeting summaries, and medication overviews	Not mentioned or unrelated to the study objective	Patient education, appointment overview, PROs feedback, and personalized activity support	Patient satisfaction, perceived knowledge and control, quality of life, and physical activity
Leader et al [56], 2021; United States	Not specified The study assesses patient portal use but does not include a detailed list of functionalities	Not mentioned or unrelated to the study objective	Not mentioned or unrelated to the study objective	Digital literacy disparities: technology access, demographic differences, and barriers to portal use
Liu et al [57], 2022; United States	Patient-accessible EHR portals Viewing test results and downloading health information to a computer or mobile device	Yes	Not mentioned or unrelated to the study objective	Patient-centered communication (interaction with health care providers), health self-efficacy (users’ confidence in managing their health), and physical and psychological health
Longacre et al [58], 2023; United States	A patient-caregiver portal system integrated within an existing patient portal Caregiver support via portal	Clinician alerts and feedback: caregiver responses are electronically shared with clinicians to inform and personalize care	Caregiver support features: patients identify caregivers, who access a personalized portal to report strain and receive tailored support resources	Usability and perceived benefits of the patient-caregiver portal system, focusing on system adoption, communication preferences, caregiver strain levels, and clinician satisfaction
Luo et al [59], 2022; United States	ePHRs^d^ broadly Access electronic health information (medical records) through patient portals	Patient-provider communication (not further specified)	Health status tracking in collaboration with health care providers (not further specified)	Factors influencing ePHR use among older cancer survivors: utilization rates, social support, confidence in security, and health-related internet use
Luoh et al [60], 2021; United States	“Epic MyChart” patient portal Patient access to test results and personal (and family) medical history	Yes	Appointment management and health maintenance monitoring	Patterns and predictors of cancer portal use, including adoption, engagement, use frequency, cancer-specific versus general use, and demographic differences
McCleary et al [61], 2018; United States	The “Dana-Farber Cancer Institute” patient portal, which is embedded within the “Epic” EHR system Access test results, including laboratory and imaging reports	Yes	Patients can access appointment schedules to manage their care and explore health and disease information relevant to their condition	Evaluation of patient portal enrollment barriers and the impact of interventions, focusing on enrollment rates after staff education, assisted enrollment, and independent enrollment support
Nahm et al [62], 2019; United States	“CaS-PET,” an interactive Cancer Survivorship Patient Engagement Toolkit Survivorship Care Plans: provide patients with detailed treatment summaries and personalized follow-up care plans.	Biweekly follow-up via portal e-messages: patients receive scheduled messages from oncology nurse navigators to assess their condition and support needs	Online survivorship resources: patients access educational modules, discussion boards, and virtual libraries through the “Well Beyond Cancer” program	The impact of “CaS-PET” on cancer survivors’ health outcomes, focusing on health-related quality of life, symptom burden reduction, patient-provider communication, and eHealth literacy
Ngo et al [63], 2020; United States	The “Personal Health Network” (PHN) mobile app is designed to support chemotherapy care coordination It includes a dashboard where patients can view components of their care plan.	Yes	A platform with a scheduling calendar, self-management library, symptom assessment surveys, and virtual meetings with caregivers and health professionals	Usability and usefulness of the PHN mobile app, focusing on patient satisfaction, care coordination benefits, and challenges related to full integration with EHR
O’Connor et al [64], 2022; United Kingdom	A portal developed on a platform and managed through the “Microsoft Azure” cloud-based system It displays prostate-specific antigen test results within hours of availability, with past results shown on a line graph for comparison over time	Yes	A patient questionnaire with clinical input option, plus prostate cancer resources (documents, videos, and links on side effects, lifestyle, and technology support)	Acceptability and usability of the patient portal: registration rates, frequency of use, satisfaction levels, and barriers to adoption
Pho et al [65], 2019; United States	“MyChart” patient portal Laboratory and tests results	Yes	Scheduling future appointments and requesting medication refills	Impact of mobile access on portal use among underserved populations, including user characteristics, access trends, and log-in frequency
Rexhepi et al [66,67], 2018, 2021; Sweden	Sweden’s national portal “Journalen” offers online EHR access, including notes, medications, laboratory results, alerts, diagnoses, referrals, and vaccines. Some portals also allow updates to personal info, record sharing, and patient-added notes.	Yes	Secure log-in, appointment booking, and prescription viewing. Includes links to trusted health resources and allows patients to store personal medical documents with their EHR.	Patients’ information-seeking via online EHRs [67]. Experiences, attitudes, and use of portals to prepare for visits; impact on empowerment and concerns about privacy and security [66].
Rexhepi et al [68], 2020; Sweden	Sweden’s “Journalen” portal provides online EHR access, including notes, medications, laboratory results, alerts, diagnoses, referrals, and vaccinations	Not mentioned or unrelated to the study objective	Not mentioned or unrelated to the study objective	Differences in EHR access attitudes and experiences between patients with cancer and those with other conditions
Santos et al [69], 2021; Canada	“MyAHS Connect” (formerly “MyChart”) was piloted in select clinics before joining Alberta’s Connect Care. It provides access to laboratory results, medications, immunizations, allergies, diagnostics, and visit notes.	Yes	Self-scheduling, medication refills, and links to trusted sources for understanding health data	Oncology patients’ and caregivers’ experiences managing care, preparing for appointments, and using health information, including awareness, adoption, and benefits
Schultz and Alderfer [70], 2018; United States	“MyNemours,” built on “Epic’s MyChart,” lets caregivers access laboratory and radiology results, diagnoses, medications, allergies, and discharge instructions.	Yes	Viewing appointments and prescription renewals online	Caregivers’ test result preferences and portal experiences, focusing on communication speed, mode, influencing factors, and perceived advantages and disadvantages
Schultz et al [71], 2021; United States	“MyNemours,” built on “Epic’s MyChart,” gives caregivers access to laboratory and radiology results, diagnoses, medications, allergies, and discharge instructions	Yes	Viewing appointments and prescription renewals online	Sociodemographic and clinical factors associated with patient portal activation among caregivers of children with cancer
Shaverdian et al [72], 2019; United States	A portal integrated into an EMR Open access to physicians’ notes (oncology notes) related to diagnosis, treatment side effects, and progress	Not mentioned or unrelated to the study objective	Not mentioned or unrelated to the study objective	Patients’ experiences with open oncology notes, including improved understanding, reassurance, and concerns like worry, confusion, or regret
Strekalova [73], 2019; United States	Focus on general EHR access through patient portals supported by United States hospitals Typically allows patients to view laboratory and test results, and summaries of past visits	Typically allows patients to send messages to health care providers	Not mentioned or unrelated to the study objective	Factors influencing portal use of patients with cancer, including demographics, behavior, perceived security and usefulness, and provider encouragement
Tarver et al [74], 2019; United States	The CRCS-PHR^e^ was developed by adapting an open-source EHR Details on cancer diagnosis, surgery, chemotherapy, and radiation therapy	Yes	Personalized side effect list, follow-up test reminders, links to support groups, and a journal for patient experiences.	Perceived usefulness of the CRCS-PHR’s medical and communication features, ease of use and satisfaction with its interface, and barriers to use
Vachon et al [75], 2022; United States	The CRCS-PHR was developed by adapting an open-source EHR Details on cancer diagnosis, surgery, chemotherapy, and radiation therapy	Not mentioned or unrelated to the study objective	Tailored side effect list, follow-up test reminders, support group links, and a journal for patient experiences	Adherence to surveillance guidelines, patient beliefs about follow-up care, and levels of self-efficacy and knowledge regarding recommended tests such as colonoscopy, carcinoembryonic antigen, and computed tomography scans
Weis et al [76], 2020; Germany	A pEHR developed for patients with cancer. Patients can grant caregivers full or graduated access to their health records. Patients and caregivers can view health-related documents.	Caregivers may share critical health information with health care providers in urgent situations	Patients can control caregiver access to their medical data, while caregivers support portal navigation, log-in, and organization of health-related documents	Caregivers’ involvement in managing the PHR, patients’ perspectives on caregiver access, challenges in granting full or limited access, and the impact on patient-caregiver relationships
Wickersham et al [77], 2019; United States	General patient portal use among cancer survivors. Access to EHRs: patients can view medications, laboratory results, visit notes, and other health data.	Yes	Patients can request prescription renewals online. Patients can authorize family members or caregivers to access their portal on their behalf.	Cancer survivors’ engagement with patient portals, adoption rates in an ambulatory cancer clinic, barriers such as provider adoption and patient motivation, and potential benefits.
Williamson et al [78], 2017; United States	A stand-alone ePHR that allows survivors to upload and store important medical records, such as Survivor Healthcare Plans, letters from oncologists, and hospital discharge notes	No e-messaging. However, users can electronically share their health documents with health care providers, regardless of institutional EMR systems.	The portal provides survivor-focused educational materials for patients and caregivers	Registration and meaningful use rates among pediatric cancer survivors, factors affecting adoption (particularly during adult care transition), and links to annual care visit adherence.
Wolff et al [79], 2019; United States	“MyChart” patient portal Patients can view test results and parts of their medical record and share access with care partners through a registration process.	Yes	Health management tasks, such as appointment scheduling	Care partner engagement in cancer communication, shared “MyChart” access impact, and changes in portal use by patients and partners.

^a^EMR: electronic medical record.

^b^PRO: patient-reported outcome.

^c^EHR: electronic health record.

^d^ePHR: electronic personal health record.

^e^CRCS-PHR: Colorectal Cancer Survivor’s Personal Health Record.

### Accessible Health Information

Regarding the access to personal health information via digital portals, the most commonly available feature was access to test and laboratory results (28/44, 64%), followed by physician notes (18/44, 41%), medication lists (15/44, 34%), and medical history, such as vaccination records (4/44, 9%).

### Availability of Secure Messaging

Regarding the availability of secure messaging, 68% (30/44) of the studies reported that this functionality was available. In 30% (13/44) of the studies, secure messaging was not mentioned, not related to the study objectives, or not applicable. One study explicitly reported that secure messaging was not available.

### Access to Health Services Provided

Regarding access to health services provided through digital portals, appointment-related functionalities such as scheduling, booking, or self-scheduling were the most frequently reported (19/44, 43%). Educational resources, general health information, or access to self-management libraries were available in 30% (13/44) of the studies, followed by medication refills, renewals, or other prescription-related features (11/44, 25%). Symptom tracking was reported in 16% (7/44) studies, caregiver access or support features in 11% (5/44) of the studies, patient-reported outcome collection in 7% (3/44) of the studies, and health status monitoring in 5% (2/44) of the studies. Access to health services was either not mentioned or not directly relevant to the study objective in 20% (9/44) of the studies.

### Assessed Outcomes

The assessed outcomes were grouped into 4 categories. Behavioral and technology experience outcomes were the most frequently reported across studies (37/44, 84%), followed by health care system-level outcomes (19/44, 43%), psychosocial outcomes (16/44, 36%), and clinical outcomes (5/44, 11%). The complete list of outcomes is presented in Textbox 1 (total number of studies reflects those that assessed at least one outcome within a given category; studies that assessed multiple outcomes within the same category are counted only once per category).

**Assessed outcomes grouped into 4 categories.**

**Behavioral and technology experience (total studies represented, n=37)**

- Portal adoption and usage behaviors (n=9)
- Self-management practices and health behaviors changes (n=7)
- User engagement (n=7)
- Perceived system usability and user-perceived benefits (n=6)
- Preferences for portal features and actual use patterns (n=5)
- Messaging frequency and email communication behavior (n=4)
- Health engagement and physical activity (n=4)
- Cancer-related portal use behaviors and content preferences (n=3)
- Access to mobile and app technologies for portal use (n=3)
- Caregiver and family member engagement, involvement, and experiences with portal use (n=3)

**Psychosocial (total studies represented, n=16)**

- Emotional responses and psychological readiness to engage with the portal (n=9)
- Patient satisfaction and subjective perceptions of portal use (n=5)
- Concerns about data security, privacy, and trust (n=4)
- Perceived psychosocial impact and quality of life (n=4)
- Patient understanding and health-related beliefs (n=3)
- Relational experiences and perceived social support (n=3)

**Clinical (total studies represented, n=5)**

- Symptom burden and control (n=4)
- Survival rates (n=1)

**Health system–level (total studies represented, n=19)**

- Demographic disparities and trends (n=10)
- Provider perspectives and engagement (n=4)
- Utilization of care (n=3)
- Access and implementation barriers (n=3)
- Enrollment and activation support (n=3)

### Associations Between PROGRESS-Plus Factors and Portal Use

The second objective was to explore the diversity of participant characteristics and potential factors associated with portal use. The PROGRESS-Plus factors [23], as interpreted by the authors of the included studies, were identified in 43% (19/44) of the studies. These factors are summarized in Table 4.

**Table 4:** Interpretation of PROGRESS-Plus (place of residence; race, ethnicity, culture, or language; occupation; gender or sex; religion; education; socioeconomic status; and social capital–Plus) factors associated with the portal use by authors of the included studies.

PROGRESS-plus factors	Authors’ interpretation
Place of residence (n=5)	Patients residing in Texas were more likely to use the portal than those living out of state [37] Rural patients with cancer were significantly less likely to email health care providers compared to urban patients [50] Patients living in areas with higher broadband access were more likely to use the portal persistently [51] Urban residents used the portal more frequently than those in rural areas [60] Those living in higher Child Opportunity Index areas were more likely to use the portal [69]
Race (or ethnicity) (n=7)	Non-Hispanic Black and Hispanic patients were significantly less likely to use e-messaging compared to non-Hispanic White patients [39] Non-Hispanic White patients were more likely to use portals than Hispanic or non-Hispanic Black patients [44] Patients “of color” logged into the portal less frequently [45] White patients had higher odds of accessing the portal compared to Black, African American, or Hispanic patients [51] Non-White patients were significantly less likely to use the portal [56] White patients were more likely to use the portal [60] White and Asian survivors were more likely to register for the portal, while Black survivors were less likely to use it meaningfully [69]
Occupation (n=1)	Employed patients were more likely to use the portal persistently [51]
Gender (or sex) (n=7)	Female patients were more likely to use the portal [35] More male patients were active users [42] Female patients were more likely to use online portals than male patients [44] A higher percentage of regular portal users were women [45] Women were more likely to access the portal than men [51] Male versus female (identified as gender by the authors) was not significantly associated with portal use [59] Male patients were more likely to use the portal [60]
Religion (n=0)	None
Education (n=6)	Higher education levels and better internet access were more likely to use the portal [35] Higher educational levels were more likely to use the portal [36] A college education or higher were more likely to use the portal [37] Active users had a higher proportion of high school education, while nonactive users had further education [42] Higher education levels were associated with increased use of portal [50] Patients with higher education levels were more likely to use the portal [56]
Socioeconomic status (n=10)	Higher household incomes were more engaged with the portal [35] No significant impact of household status on the portal use [36] Middle-income earners (US $30,000-$99,999) were more frequent users compared to higher-income earners [37] The patients with managed care were more likely to use e-messaging compared to those with Medicare or Medicaid [39] Higher income levels were linked to more frequent use of portal [50] Income not significantly linked to portal use [59] Patients with private insurance had higher use rates [60] Those with higher socioeconomic status were more likely to use the portal [64] Those with private health insurance were more likely to use the portal [69] Those with higher income levels were more likely to use portals frequently [73]
Social capital (n=2)	All active users lived with someone, while nonactive users included those living alone [42] Participants with more social support experienced lower odds of using portals [59]
Age (n=13)	Older patients were more likely to use the portal [35] No significant age difference between users and nonusers [36] Younger patients were more likely to use e-messaging [39] Active users were slightly younger on average (44.3 y) compared to nonactive users (49.2 y) [42] Older patients (≥65 y) were less likely to use portals compared to younger patients [44] Younger patients logged into the portal less frequently [45] Older patients were less likely to use email to communicate with their health care providers [50] Younger patients (<40 y) were more likely to access the portal compared to older patients (>65 y) [51] Younger patients were more likely to use the portal [56,60] Older patients were less likely to enroll in the portal [61] Older patients with prostate cancer were less likely to register and use the portal [64] Younger children had higher odds of their caregivers activating the portal [69]
Disability (n=1)	Greater physical impairment was associated with higher portal use [37]
Other vulnerabilities (n=6)	Health literacy Higher health literacy felt more comfortable navigating and understanding the portal [35] Language English-speaking patients were more likely to use e-messaging, and those requiring an interpreter were less likely to use it [39] Caregivers who spoke English were significantly more likely to activate the portal [69] Technical proficiency Active users generally had better computer and internet skills [42] Computer access Patients without computer access were less likely to enroll in the portal [61] Information technology skills Lack of computer skills and access to computing facilities were common reasons for nonuse [64]

Among the PROGRESS-Plus factors, age was the most frequently reported dimension, addressed in 68% (13/19) of the included studies. This was followed by socioeconomic status (10/19, 53%), and both race or ethnicity and gender or sex, each included in 37% (7/19) of the studies. In contrast, social capital was reported in only 11% (2/19) of the studies, while occupation and disability were each addressed in 5% (1/19) of the studies. Religion was not represented in any of the included studies.

In addition to the PROGRESS-Plus factors, we identified 5 individual, cancer-related characteristics associated with the portal use (Textbox 2 [42,45,60,69,78]).

**Individual, cancer-related characteristics associated with portal use.**

- Individuals with bone cancer and those in the active treatment phase were more likely to use the portal [42].
- Each additional oncology office visit in a month increased the frequency of portal log-ins [45].
- Individuals with metastatic cancer were more frequent users compared to those with nonmetastatic cancer [60].
- Caregivers of children undergoing longer treatments, and more radiology tests were more likely to activate the portal [69].
- Those who transitioned from pediatric to adult care used the portal more consistently and frequently [78].

## Discussion

### Principal Findings

Most of the included studies were conducted in the United States, reflecting the widespread implementation of patient portals with interoperable features in that country during the early 2010s [49,65]. Common portal functionalities, such as those offered by “MyChart,” developed by “Epic Systems,” include access to laboratory and test results, secure messaging with clinical teams, appointment scheduling, and prescription refill requests. These features appear to have shaped the focus of the studies included in this review.

The outcomes assessed aligned with the available portal functionalities. Behavioral and technology experience outcomes, psychosocial outcomes, and health system–related outcomes were assessed more frequently than clinical outcomes. Symptom tracking, patient-reported outcome collection, and health status monitoring were less commonly described. None of the studies reported features that allowed patients to add or amend notes in their medical records. The use of virtual or remote consultations was explicitly specified in only 2 studies [41,43].

Only 4 studies in our review focused on symptom-related clinical outcomes [37,41,46,62]. While confounding factors limit causal inference, these studies highlight portal features that may facilitate symptom management. Identified functionalities included access to educational resources [37], electronic symptom tracking [41], symptom and distress monitoring [46], and personalized care planning with scheduled follow-up messaging by oncology nurses [62]. Structured follow-up, individualized education, and active monitoring appear particularly promising. These features warrant greater integration into portals and further investigation to better understand their potential impact on symptom burden and overall clinical outcomes.

Regarding the diversity of participant characteristics and potential factors associated with portal use, the evidence was heterogeneous. Age was frequently examined, but the findings were inconsistent. Some studies reported greater portal use among younger individuals [42,51,56,60], while others observed higher use among older adults [35,45]. Gender-related findings were similarly mixed: in some cases, women were more likely to use portals [35,44,45,51], while in others, men were [42,60]. All studies assessed gender in binary terms, comparing men and women only; none of the studies included gender-diverse identities.

Other PROGRESS-Plus factors demonstrated more consistent associations. In studies conducted in the United States, White and Asian participants were generally more likely to use portals than Black or Hispanic participants [39,44,45,51,56,60,78]. Similarly, individuals with higher socioeconomic status [35,39,50,60,64,69,73] and those residing in urban areas [37,50,51,60,69] were generally more likely to engage with portals than those living in rural settings. In contrast, factors such as social capital, occupation, disability, and religion were rarely explored. Additional vulnerability-related characteristics were also identified, including language spoken [39,69], access to computers [61,64], health literacy [35], and digital proficiency [42,64]. These factors may influence equitable access to and use of patient portals.

### Comparison With Previous Work

We identified 3 reviews that examined patient portals among populations with various health conditions [1,5,7]. In addition, 3 reviews explored digital health interventions for individuals living with or beyond cancer, although they did not focus specifically on patient portal use [3,8,22]. Our review adds to this body of work by focusing exclusively on individuals with or beyond cancer and their use of portals, defined as an access to personal health information or data [1,2,4-7].

One previous review, published in 2018, specifically addressed portal use among individuals with cancer [4]. It concluded that portals may support self-management, a behavioral outcome, particularly among individuals beyond cancer. Consistent with our findings, portal use was more common among White individuals and those with higher socioeconomic status. While that review called for further research on factors influencing portal use, our work provides an updated synthesis that incorporates the PROGRESS-Plus factors framework and captures a broader range of outcomes.

Another review of portal functionalities for individuals with diabetes reported that half of the included studies (6 out of 12) featured secure messaging, and a smaller portion (2 out of 12) provided access to health services [1]. These proportions were lower than what we observed in our review. In contrast to our findings, which included few clinical outcome assessments, that review identified associations between portal use and improved glycemic control. Similarly, another review examining portal use across diverse populations found that while behavioral outcomes were generally positive, the effects on clinical outcomes remained inconsistent, likely due to confounding factors [5].

One review focusing on patient education delivered through portals reported increased user engagement, improved behavioral outcomes, and high levels of satisfaction [7]. These results align with our findings, which indicate a stronger focus on behavioral and technology experience outcomes. In a breast cancer population, a review of eHealth tools, including portals, found mixed effects on symptoms and lifestyle-related outcomes, although user satisfaction was generally high [8]. Another review of digital health technologies also reported improvements in behavioral outcomes and technology-related experiences, particularly in the context of clinician-patient communication [3].

In relation to PROGRESS-Plus factors, a review on patient-centered technologies for underserved cancer populations in the United States, including African American, Hispanic, and rural communities, reported improved behavioral outcomes, such as better screening adherence and increased cancer-related knowledge [22]. These populations remain underrepresented in digital health research, reinforcing the relevance of our equity-focused analysis.

Prior reviews also identified several barriers to effective portal use. This included difficulty navigating complex interfaces and limited support for certain populations, particularly those with lower digital literacy [22]. In addition, a review on oncology portal use noted that while many patients accessed their health records, they often struggled to interpret the information they found [4].

Together, these findings are consistent with our review and support the need for more inclusive, user-centered portal design. Tailored implementation strategies that address the needs of diverse populations are important to ensuring equitable access and meaningful engagement, particularly when considering the PROGRESS-Plus factors identified in our review.

### Strengths and Limitations

This review has several strengths. First, 44 studies exploring the use of digital health portals among individuals living with or beyond cancer were identified. Our inclusion criteria extended beyond portals solely tethered to medical records, encompassing all digital platforms that enabled these individuals to access their personal health information or data. Second, we identified and categorized portal functionalities into 3 distinct categories, and we grouped outcomes into 4 categories. Third, we applied the PROGRESS-Plus framework to identify potentially underserved populations and to highlight actionable opportunities for promoting health equity.

Nonetheless, some limitations should be acknowledged. First, we limited our search to studies published in the 10 years preceding March 2024. This time frame was selected to reflect current technological capabilities and patient engagement practices, with an emphasis on more advanced and interoperable portal systems. Given the pace of technological change during this period, it is unlikely that major relevant studies were overlooked. Second, our search strategy was not peer-reviewed by an independent librarian. However, detailed documentation is provided in Multimedia Appendix 1 to support transparency and replicability. Third, data extraction was conducted once by 4 novice reviewers. To ensure accuracy and consistency, all extracted data were subsequently validated by the first author (SO) and an experienced reviewer (MS) with expertise in methodology and digital health technologies.

### Conclusions

This review provides an overview of digital health portal use among individuals living with or beyond cancer, encompassing both patient portals and PHRs. While these tools are increasingly implemented to support patient self-management, their actual impact on clinical outcomes remains uncertain. Our findings indicate that research has predominantly focused on portals implemented in the United States and has emphasized behavioral and technology experience outcomes, with comparatively limited attention to clinical outcomes and equity considerations.

Disparities were observed in the availability of portal functionalities, the types of outcomes assessed, and the extent to which PROGRESS-Plus factors were reported or analyzed. Features such as secure messaging and access to services such as appointment scheduling and medication renewals were the most described. In contrast, functionalities such as personalized care programs and symptom tracking tools were less frequently represented. Furthermore, portal use was lower among certain population groups, and several PROGRESS-Plus factors remained underexplored or absent from analysis.

These findings offer valuable insights for researchers, health care providers, policy makers, patient advocacy groups, and digital health engineering teams engaged in the design and implementation of patient-centered technologies. To ensure that digital health portals contribute meaningfully to cancer care for all individuals, future research should prioritize more inclusive designs and evaluation strategies that address both outcome diversity and social determinants of health.

## Appendix

Multimedia Appendix 1 Search strategies by database.

Multimedia Appendix 2 Number of articles published per year (2014 to 2024).

Multimedia Appendix 3 Participant cancer types and stages.

Multimedia Appendix 4 PRISMA-ScR checklist.

## Abbreviations

EHR: electronic health record
EMR: electronic medical record
PCC: population (or participant), concept, and context
PHR: personal health record
PRISMA: Preferred Reporting Items for Systematic reviews and Meta-Analyses
PRISMA-ScR: Preferred Reporting Items for Systematic reviews and Meta-Analyses extension for Scoping Reviews
PROGRESS-Plus: place of residence; race, ethnicity, culture, or language; occupation; gender or sex; religion; education; socioeconomic status; and social capital–Plus

## Data Availability

The database search strategies are available in Multimedia Appendix 1. A table of participant cancer types and stages is provided in Multimedia Appendix 3. The PRISMA-ScR (Preferred Reporting Items for Systematic Reviews and Meta-Analyses extension for Scoping Reviews) checklist can be found in Multimedia Appendix 4. All data generated or analyzed during this study are included in this published article and its supplementary information files.
