# Supplementary material for: Digital Health Portals for Individuals Living With or Beyond Cancer: Patient-Driven Scoping Review
Source: JMIR Cancer. 2025 Jul 18;11:e72862. doi: 10.2196/72862 (PMC12317290; doi:10.2196/72862)
Supplement: Multimedia Appendix 1 [file cancer_v11i1e72862_app1.pdf]

The search strategies for each database

CINAHL Plus with Full Text (EBSCO)

Date of the search: 2024-02-27

Database limit: 2014 -Current

#	Search strategy	Results
1	TI (((Patient* N2 portal*) NOT (vein OR venous OR "portal hypertension" OR liver))) OR AB (((Patient* N2 portal*) NOT (vein OR venous OR "portal hypertension" OR liver)))	1,253
2	TI ("health* portal*" OR "online portal*" OR "Patient platform*") OR AB ("health* portal*" OR "online portal*" OR "Patient platform*")	300
3	TI ((((web OR "web based") N2 portal*) AND (patient* OR personal))) OR AB ((((web OR "web based") N2 portal*) AND (patient* OR personal)))	284
4	TI (("electronic portal*" AND (patient* OR personal) NOT Imaging)) OR AB (("electronic portal*" AND (patient* OR personal) NOT Imaging))	60
5	TI ("personal health record*" OR "personal medical record*" OR "personal health information management system*") OR AB ("personal health record*" OR "personal medical record*" OR "personal health information management system*")	769
6	(MH "Patient Portals")	336
7	((MH "Electronic Health Records") OR TI "electronic medical record*" OR AB "electronic medical record*") AND ((MH "Patient Access to Records") OR TI patient* N2 access* OR AB patient* N2 access* OR TI personal OR AB personal)	1,633
8	S1 OR S2 OR S3 OR S4 OR S5 OR S6 OR S7	3,781
9	TI (Cancer OR Oncolog*) OR AB (Cancer OR Oncolog*)	534,213
10	(MH "Neoplasms+") OR (MH "Cancer Patients") OR (MH "Cancer Survivors") OR (MH "Cancer Care Facilities") OR (MH "Oncology+")	701,66
11	S9 OR S10	854,339
12	S8 AND S11	332
13	S8 AND S11 Restriction operators - Publication date: 20140101-20241231	266

Web of Science (A&HCI , ESCI ,SCI-EXPANDED , SSCI)
Date of the search: 2024-02-26
Database limit: 2014 -Current

#	Search strategy	Results
1	TS=(((Patient* NEAR/2 portal*) NOT (vein OR venous OR "portal hypertension" OR liver))) Editions: WOS.SCI,WOS.SSCI,WOS.AHCI,WOS.ESCI	2 511
2	TS=("health* portal*" OR "online portal*" OR "Patient platform*") Editions: WOS.SCI,WOS.SSCI,WOS.AHCI,WOS.ESCI	944
3	TS=((((web OR "web based") NEAR/2 portal*) AND (patient* OR personal))) Editions: WOS.SCI,WOS.SSCI,WOS.AHCI,WOS.ESCI	734
4	TS=(("electronic portal*" AND (patient* OR personal) NOT Imaging)) Editions: WOS.SCI,WOS.SSCI,WOS.AHCI,WOS.ESCI	151
5	TS=("personal health record*" OR "personal medical record*" OR "personal health information management system*") Editions: WOS.SCI,WOS.SSCI,WOS.AHCI,WOS.ESCI	1 664
6	TS=("electronic medical record*" AND ((patient* NEAR/2 access*) OR Personal)) Editions: WOS.SCI,WOS.SSCI,WOS.AHCI,WOS.ESCI	990
7	#6 OR #5 OR #4 OR #3 OR #2 OR #1 Editions: WOS.SCI,WOS.SSCI,WOS.AHCI,WOS.ESCI	6 160
8	TS=(Cancer OR Oncolog*) Editions: WOS.SCI,WOS.SSCI,WOS.AHCI,WOS.ESCI	3 279 907
9	#8 AND #7 Editions: WOS.SCI,WOS.SSCI,WOS.AHCI,WOS.ESCI	770
10	#8 AND #7 Editions: WOS.SCI,WOS.SSCI,WOS.AHCI,WOS.ESCI Timespan: 2014-01-01 to 2024-12-31	593

Medline (Ovid)
Date of the search: 2024-02-26
Database limit: 2014 -Current

#	Search strategy	Results
1	((Patient* adj3 portal*) not (vein or venous or "portal hypertension" or liver)).ab,kf,ti.	2515
2	("health* portal*" or "online portal*" or "Patient platform*").ab,kf,ti.	657
3	(((web or "web based") adj3 portal*) and (patient* or personal)).ab,kf,ti.	688
4	(("electronic portal*" and (patient* or personal)) not Imaging).ab,kf,ti.	144
5	("personal health record*" or "personal medical record*" or "personal health information management system*").ab,kf,ti.	1449
6	Patient Portals/	799
7	(Electronic Health Records/ or "electronic medical record* ".ab,kf,ti.) and (Patient Access to Records/ or (patient* adj3 access*).ab,kf,ti. or personal.ab,kf,ti.)	2523
8	1 or 2 or 3 or 4 or 5 or 6 or 7	6749
9	(Cancer or Oncolog*).ab,kf,ti.	2319428
10	exp Neoplasms/ or Cancer Survivors/ or Cancer Care Facilities/	3940776
11	9 or 10	4590928
12	8 and 11	840
13	limit 12 to yr="2014 -Current"	591

Embase (embase.com)
Date of the search: 2024-02-26
Database limit: 2014 -Current

#	Search strategy	Results
1	((patient* NEAR/3 portal*):ti,ab,kw) NOT (vein:ti,ab,kw OR venous:ti,ab,kw OR 'portal hypertension':ti,ab,kw OR liver:ti,ab,kw)	3829
2	'health* portal*':ti,ab,kw OR 'online portal*':ti,ab,kw OR 'patient platform*':ti,ab,kw	1195
3	(((web OR 'web based') NEAR/3 portal*):ti,ab,kw) AND (patient*:ti,ab,kw OR personal:ti,ab,kw)	1323
4	'electronic portal*':ti,ab,kw AND (patient*:ti,ab,kw OR personal:ti,ab,kw) NOT imaging:ti,ab,kw	259
5	'personal health record*':ti,ab,kw OR 'personal medical record*':ti,ab,kw OR 'personal health information management system*':ti,ab,kw	1716
6	'patient portal'/de	10
7	('patient right'/de OR ((patient* NEAR/3 access*):ti,ab,kw) OR 'personal':ti,ab,kw) AND ('electronic health record'/de OR 'electronic medical record*':ti,ab,kw OR 'electronic health record*':ti,ab,kw)	4607
8	#1 OR #2 OR #3 OR #4 OR #5 OR #6 OR #7	11197
9	cancer:ti,ab,kw OR oncolog*:ti,ab,kw	3404913
10	'neoplasm'/exp OR 'cancer center'/de OR 'cancer patient'/exp OR 'oncology'/exp	6313843
11	#9 OR #10	6807774
12	#8 AND #11	2160
13	#8 AND #11 AND [2014-2024]/py	1732
