# Supplementary figures and images for: Digital Health Portals for Individuals Living With or Beyond Cancer: Patient-Driven Scoping Review
Source: JMIR Cancer. 2025 Jul 18;11:e72862. doi: 10.2196/72862 (PMC12317290; doi:10.2196/72862)

**Number of articles published per year (2014 to 2024)**
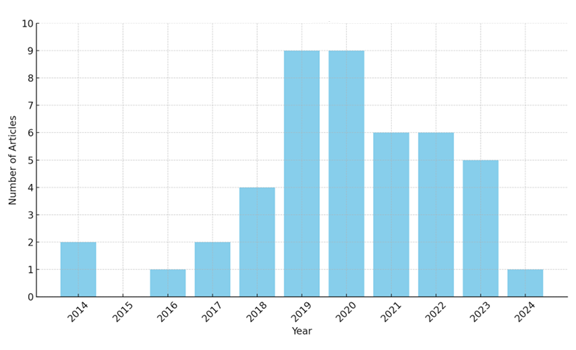

Supplement: Multimedia Appendix 2 [file cancer_v11i1e72862_app2.docx]
