# Supplementary material for: Digital Health Portals for Individuals Living With or Beyond Cancer: Patient-Driven Scoping Review
Source: JMIR Cancer. 2025 Jul 18;11:e72862. doi: 10.2196/72862 (PMC12317290; doi:10.2196/72862)
Supplement: Multimedia Appendix 3 [file cancer_v11i1e72862_app3.pdf]

Participant Cancer Types and Stages

**First author, year, country**	**Number of participants**	**Types of cancer among participants *Indicate when discussing metastasis; otherwise, omit mention.***
Alpert, 2019, USA [35]	35 patients, *13 oncologists, and 12 informaticists*.	The most common conditions were breast cancer (24%), hematologic (16%), and gastrointestinal (13%). Less than half (15/35) of patients had metastatic cancer and about a third (34%) had Stages 2–4 cancer. 46% of the participants had stage IV cancer, which indicates the presence of metastases.
Baun, 2020, Denmark [36]	Questionnaire: 46 patients; Interviews: 4 patients.	Women with metastatic breast cancer.
Cahill, 2014 USA [37]	186 patients.	Brain tumor patients: Glioblastoma/Gliosarcoma: 44%, Astrocytoma: 24%, Oligodendroglioma: 21%, Oligoastrocytoma: 4%, Ependymoma: 2%
Colussi, 2024, Argentina [38]	422 responses (a person could have answered the survey more than once).	Various types of cancer, such as breast cancer, prostate cancer, and blood cancer.
Conroy, 2023, USA [39]	4,069 patients.	Breast cancer not further specified: patients who completed a visit at an academic breast center and sent at least one message to a provider during the study period (May 2021-May 2022).
Coquet, 2020, USA [40]	After propensity score matching, the analysis focused on 6,446 patients: 3,223 email users and 3,223 non-email users.	Included cancer patients undergoing chemotherapy for various types of cancer. Primary cancer types among the participants: Breast Cancer, Lung Cancer, Prostate Cancer, etc.
Daly, 2020, USA [41]	100 patients.	Thoracic cancer: 30%, Gastrointestinal (GI): 22%, Head and Neck: 22%, Gynecologic: 8%, Genitourinary: 8%, Breast: 7%, Lymphoma: 3% / 39% of the enrolled patients had a high tumor burden or a site of metastasis.
DeRegge, 2020, Belgium [42]	23 patients.	Two types of cancer:<br>Bone Cancer: 16 patients<br>Kidney Cancer: 7 patients

		(13 out of the 23 participating patients had metastases).
Ector, 2020, Netherlands [43]	8 patients (*and 14 hematologists*).	Patients with chronic myeloid leukemia (CML).
Elkefi, 2021, USA [44]	4,328 patients.<br>693 with cancer.	Patients (N = 4,328) having experienced an outpatient visit within the previous 12 months.<br>16% (693 patients) of the respondents had been diagnosed with cancer).
Emamekhoo, 2023, USA [45]	2,076 patients.	Breast Cancer: 26% of participants,<br>Colon and Rectum Cancer: 8.8%,<br>Lung: 12%, Prostate: 4.8%, Other Types: 49% of participants.<br>Among the regular portal users, 46% had metastatic cancer, while 54% had nonmetastatic cancer.
Fridriksdottir, 2023, Iceland [46]	69 patients.	Patients with gastrointestinal and hematological cancers.<br>Included patients with various stages of cancer, including those with stage IV cancer, which indicates metastases. Specifically, 46% of the patients had stage IV cancer.
Geerts, 2019, Netherlands [47]	204 patients (*and 13 physicians*).	Only mentioned that the patients have a haematologic malignancy.
Geerts, 2023, Netherlands [48]	18 patients.	Patients with multiple myeloma (MM), a type of blood cancer.
Gerber, 2014, USA [49]	6,495 patients.	20% had breast cancer, 16% gastrointestinal/genitourinary, 12% hematologic, 7% upper aerodigestive, and 33% other types.
Greenberg-Worisek, 2020, USA [50]	3,031 cancer patients/ survivors.	Only mentions that the survey included individuals who reported having been diagnosed with cancer, including skin cancers.
Griffin, 2023, USA [51]	28,942 patients.	Specific details about the types of cancer not provided.
Groen, 2017, Netherlands [52]	37 patients.	Patients with non-small cell lung cancer.
Haggstrom, 2022, USA [53]	6 patients (and 4 caregivers).	Colorectal cancer (CRC) survivors.
Kayastha, 2018, USA [54]	20 patients.	Various types of metastatic or incurable cancer: Colon: 4 participants. Breast: 3 participants.<br>Lung/Bronchus: 2 participants. Prostate: 2 participants. Pancreas: 2 participants.<br>Adenocarcinoma of unknown primary site: 2 participants.<br>Other types: 5 participants.

Kuijpers, 2016, Netherlands [55]	92 patients.	Histologically confirmed breast cancer: Ductal Carcinoma In Situ (DCIS): 11%. Stage I: 28.6%. Stage II: 45.1%. Stage III: 15.4%.
Leader, 2021, USA [56]	346 patients (and 13 caregivers).	Patients with cancer and caregivers: Types of cancer not specified.
Liu, 2022, USA [57]	626 patients.	Cancer survivors: types of cancer not specified.
Longacre, 2023, USA [58]	20 patients and 19 caregivers.	Kidney Cancer: 20% of patients. Lung Cancer: 20% of patients. Breast Cancer: 15% of patients. Other types included endometrial, leukemia, lymphoma, melanoma, multiple myeloma, ovarian, pancreatic, and thyroid cancers. Most had late-stage cancer (70% stage IV).
Luo, 2022, USA [59]	207 patients.	Older cancer survivors: types of cancer not specified.
Luoh, 2021, USA [60]	5,950 patients.	Breast: 21.6% of participants. Lung: 13.1% of participants. Colon and Rectum: 7.3% of participants. Prostate: 4.8% of participants. Other Cancers: 48.7% of participants. 1,256 patients with metastatic cancer (21.1% of the 5,950 patients).
McCleary, 2018, USA [61]	1,019 patients. Focus groups: (staff n=20; patient representatives, n=5)	Breast: 20%. Gastrointestinal: 20%. Hematologic Oncology: 17%. Other Types: Including bone and sarcoma, gynecologic malignancies, head and neck oncology, melanoma, neuro-oncology, thoracic oncology, etc.
Nahm, 2019, USA [62]	30 patients.	Cancer survivors: types of cancer not specified.
Ngo, 2020, USA [63]	27 patients.	Types of cancer not specified: all participants had a primary diagnosis of cancer (any site) and were initiating chemotherapy.
O'Connor, 2022, UK [64]	518 patients.	Prostate cancer.
Pho, 2019, USA [65]	2,524 patients.	Oncology patients: types of cancer not specified.
Rexhepi, 2018, and Rexhepi, 2021, Sweden [66,67]	30 patients.	Participants in different stages of cancer, including newly diagnosed, recurrence, and advanced stages receiving palliative treatment.
Rexhepi, 2020, Sweden [68]	347 (out of 2,587) respondents with cancer.	Patients with different diseases using an online portal. Types of cancer of the 347 patients not specified.

Santos, 2021, Canada [69]	10 patients (and 1 family caregiver).	Various types of cancer, including blood-borne, tissue, organ, or lymphatic cancer.
Schultz, 2018, USA [70]	19 caregivers of children.	Leukemia/lymphoma: 7. Ewing sarcoma: 3. Wilms tumor: 2. Germ cell tumor: 2. Brain tumor: 2. Osteosarcoma: 1. Neuroblastoma: 1. Rhabdomyosarcoma: 1.
Schultz, 2021, USA [71]	390 caregivers of children.	Liquid cancers: These include cancers originating from bone marrow, such as leukemia: 90 (28%).<br>Solid cancers: These include cancers originating from organs or tissues, such as rhabdomyosarcoma and Hodgkin lymphoma: 232 (72%).
Shaverdian, 2019, USA [72]	136 patients (baseline survey completed)	Cancer Diagnosis: The most common diagnoses were breast cancer (24%), head and neck (21%), lung (16%), prostate (13%), and other types (27%).
Strekalova, 2019, USA [73]	542 patients.	A sample including individuals who have had a cancer diagnosis.
Tarver, 2019, USA [74]	22 patients.	Colorectal cancer (CRC) survivors.<br>The study excluded patients with metastatic disease.
Vachon, 2022, USA [75]	28 patients (baseline survey completed).	Colon Cancer: 32% of the participants. Rectal Cancer: 64% of the participants. One participant's cancer type was unknown. The study excluded patients with metastatic disease.
Weis, 2020, Germany [76]	22 patients (and 9 caregivers).	The participants were patients with gastrointestinal tumor diseases, specifically diagnosed with: Colorectal, Stomach, and Gallbladder cancer. These patients were receiving chemotherapy.
Wickersham, 2019, USA [77]	85 patients.	The most common types: Leukemia/Lymphoma: 32.1%. Multiple Myeloma: 23.5%. Breast Cancer: 4.9%. Lung Cancer: 4.9%.
Williamson, 2017, USA [78]	624 pediatric cancer survivors.	Leukemia was the most common diagnosis (45%), followed by sarcoma (13.8%), neuroblastoma (10.7%), renal tumors (9.3%), non-Hodgkin's (9%), Hodgkin's lymphoma (6.1%), and other malignancies (6.1%).
Wolff, 2019, USA [79]	132 patients and family care partners dyads.	Patients undergoing active treatment for breast cancer. The study focused on patients with either early stage or advanced breast cancer. Nearly half of the patients (45.5%) in the study were diagnosed with metastatic disease.
